# Supplementary material for: A high-resolution mRNA expression time course of embryonic development in zebrafish
Source: eLife. 2017 Nov 16;6:e30860. doi: 10.7554/eLife.30860 (PMC5690287; doi:10.7554/eLife.30860)
Supplement: Supplementary file 6. [file elife-30860-supp6.zip › biolayout-clusters-files/Cluster180-genes.html]

Cluster180


# Cluster180: Genes

| Ensembl ID | Gene Name | Chr | Start | End | Biotype |
| --- | --- | --- | --- | --- | --- |
| ENSDARG00000068166 | dfnb31b | 5 | 28403743 | 28522462 | protein\_coding |
| ENSDARG00000059340 | dlgap2a | 17 | 24917658 | 25157159 | protein\_coding |
| ENSDARG00000074149 | itpr1b | 11 | 35515344 | 35758022 | protein\_coding |
| ENSDARG00000035360 | mink1 | 5 | 37705420 | 37784602 | protein\_coding |
| ENSDARG00000077710 | nlgn1 | 11 | 9579607 | 9589562 | protein\_coding |
| ENSDARG00000042410 | rab40b | 12 | 31924275 | 31951568 | protein\_coding |
| ENSDARG00000098438 | slc6a6b | 22 | 31584965 | 31600386 | protein\_coding |
| ENSDARG00000061819 | wscd2 | 5 | 20123400 | 20174500 | protein\_coding |
